# Should I sit or stand: likelihood of adherence to messages about reducing sitting time

**DOI:** 10.1186/s12889-019-7189-z

**Published:** 2019-07-03

**Authors:** Stephanie J. Alley, Corneel Vandelanotte, Mitch J. Duncan, Camille E. Short, Jaclyn P. Maher, Stephanie Schoeppe, Amanda L. Rebar

**Affiliations:** 1 Physical Activity Research Group, Appleton Institute, School of Health, Medical and Applied Sciences, Central Queensland University, Building 7, Bruce Hwy, Rockhampton, QLD 4701 Australia; 2 School of Medicine & Public Health, Priority Research Centre for Physical Activity and Nutrition, Faculty of Health and Medicine, The University of Newcastle, Callaghan, NSW Australia; 3 School of Psychological Sciences and Melbourne School of Health Sciences, Melbourne University, Melbourne, VIC Australia; 4 Department of Kinesiology, University of North Carolina, Greensboro, NC USA

**Keywords:** Health promotion, Campaign, Mass media, Sitting time, Sedentary behaviour, Behaviour change

## Abstract

**Background:**

High population levels of sitting is contributing to high rates of chronic health problems. Therefore, the aim of this study was to identify the sitting time messages with the greatest potential to reduce sitting behaviour, as well as identify how this may differ according to demographic, behavioural and psychosocial characteristics.

**Methods:**

Australian adults (*N* = 1460) were asked to report the likelihood that they would adhere to seven messages promoting reduced sitting time and two messages promoting increased physical activity (from ‘not at all likely’ to ‘very likely’). Ordinal regression models were used to compare messages on the likelihood of adherence and whether likelihood of adherence differed as a function of demographic, psychosocial and behavioural characteristics.

**Results:**

Likelihood of adherence was highest for the messages, ‘Stand and take a break from sitting as frequently as you can’ (83% respectively) and ‘Avoid sitting for more than 10 hours during the entire day’ (82%) and was significantly lower for the message, ‘Sit as little as possible on all days of the week’ (46%) compared to all other messages.

**Conclusions:**

To increase likelihood of adherence messages should be specific, achievable and promote healthy alternatives to sitting (e.g. standing). Messages promoting standing as a healthy alternative to sitting may be more likely to engage people with high sitting behaviour and messages promoting physical activity may be more likely to engage males and retired adults.

**Electronic supplementary material:**

The online version of this article (10.1186/s12889-019-7189-z) contains supplementary material, which is available to authorized users.

## Background

Research indicates that prolonged sitting (8+ hours per day) leads to increased risk of chronic disease and mortality [1–3]. Studies also show that interrupting periods of sitting with short light- or moderate-intensity walking can improve cardio-metabolic risk factors [4]. Health departments worldwide including Australia, France and the UK have targets to reduce sedentary behaviour and promote limiting sitting time [5–7]. In Australia, adults are encouraged to minimise the amount of time spent in prolonged sitting and to break up long periods of sitting as often as possible [5]. Despite this over 25% of Australians sit at least 8 h per day [3, 8].

Public health recommendations can effectively be distributed to large populations as concise messages disseminated through mass media campaigns [9]; however, the effectiveness of these efforts differ based on content and framing of the message [10, 11]. For example, meta-analyses of mass media campaigns promoting health behaviours such as dental hygiene and safe sex found that behaviour change was greater for positively framed messages about the adoption of new health behaviours compared to negatively framed messages about the reduction of unhealthy behaviours [12]. As such, people may be more likely to adhere to messages that promote standing or physical activity (as a replacement to sitting) compared to messages that promote the avoidance of sitting. However, no research has specifically investigated what framing and content (e.g. setting, specificity etc.) of healthy sitting messages is the most effective at engaging the public.

Particular message framing styles and content can be better suited to people with certain demographic characteristics. For example, positively framed physical activity messages have been found to be particularly effective in older males [13–15]. Behavioural (e.g., current sitting time and physical activity) characteristics may also affect people’s likelihood of adhering to the messages, as it affects the extent to which they need to change their behaviour (e.g., it is easier to reach less than 10 h sitting a day if currently doing 11 h compared to 15 h). Lastly psychosocial (e.g., knowledge of sitting risk and intentions to decrease sitting) characteristics may also affect people’s likelihood of adhering to the messages. The Theory of Planned Behaviour stipulates that attitudes (knowledge of benefits and barriers to performing a behaviour), social norms (support and modelling from others) and perceived behavioural control (confidence to perform a behaviour) influence intentions, which in turn influence behaviour [16]. The Theory of Planned Behaviour is a good predictor of health behaviours and is commonly used to inform health behaviour change interventions [17, 18]. Specifically, knowledge of health risks can contribute to attitudes of the target behaviour [19] which influences intentions and behaviour [17, 20, 21]. Intentions to change a behaviour does not always lead to behaviour change, a phenomenon which is termed the intention-behaviour gap. However, intentions to change behaviour is a strong and consistent predictor of behaviour change [17, 20, 21]. Therefore knowledge and intentions about sitting time may influence people’s likelihood of adhering to messages to reduce sitting time. However, no research has investigated which framing styles and content of sitting messages are suited to different population groups. Therefore, research on how sitting messages are received by different population groups is required to inform targeted mass media campaigns.

The purpose of this study was to examine adults’ impressions of different messages about reducing sitting time. The first objective was to examine a number of healthy sitting and physical activity messages for self-reported likelihood of adherence which is a predictor for behaviour change [22–24]. The second objective was to determine whether self-reported likelihood of message adherence differed as a function of demographic (age, gender, education, employment, disease status), behavioural (sitting time and physical activity) and psychosocial (knowledge and intentions) characteristics.

## Method

This study was part of the Australian Health and Social Science panel project. For the panel project, adults were recruited to be panel members annually in 2009–2012 through computer-assisted phone calls made to randomly selected households in each state of Australia. People who were over 18, resided in Australia and could speak and read the English language were eligible to participate. Panel members completed an initial online demographic survey, then were invited via email to participate in regular online surveys. A total of 3932 people became panel members. More detail about the panel project can be found elsewhere [25]. Panel members were invited to complete the web-based survey for the current study via email during August and September 2012, and it was completed by 1843 panel members (42% response rate). Informed consent was obtained from all participants and study procedures were approved by Central Queensland University Human Research Ethics Committee (H12/06–126). Data from participants who reported they had a health condition that prevented them from decreasing their sitting time (*n* = 195, 11%) or had missing data (*n* = 188, 10%) were omitted from the analyses. The final sample size was *N* = 1460.

### Measures

#### Demographic characteristics

Participants reported their gender, education (year 12 or less, advanced degree), employment status (full time, part time, retired, unemployed), and age. Participants were also provided with a list of 24 chronic health conditions (e.g., asthma, coronary heart disease, dementia) and asked to tick all the diseases they had been diagnosed with by a clinician. Disease status was coded as having one or more of the listed chronic conditions or none of the listed chronic conditions.

#### Health behaviours

Sitting time was assessed with the valid and reliable 10-item Workforce Sitting Questionnaire [26]. Participants reported how much time they spent sitting in leisure, working, transport, computer, and television contexts during the past 7 days. Total sitting time on a workday and a non-workday was defined as the sum of sitting time in all domains on a workday and a non-workday respectively. In addition, participants reported the number of days they were at work in the last 7 days, and this was used to calculate average total sitting time per day. Extreme values (*n* = 32, 2%) were truncated (*M* + 3*SD) to reduce the impact of outliers.

Physical activity was assessed through the Active Australia Survey which has acceptable validity and reliability [27, 28]. Participants reported the total time they spent walking continuously for 10 min or longer and engaged in moderate (e.g., gentle swimming, social tennis, golf) and vigorous (e.g., jogging, cycling, aerobics, competitive tennis) activity in the past week. Minutes of physical activity per week was calculated as the sum of the time spent walking, time in moderate activity and time in vigorous activity (weighted by two to account for the extra energy expenditure output) as per manual instructions [27].

#### Intentions

Intentions to limit sitting were assessed using items from the Theory of Planned Behaviour questionnaire [29], and were constructed to align with the Workforce Sitting Questionnaire [26]. Participants were asked “Do you intend to sit less than you do now. ..” in the five different contexts assessed in the Workforce Sitting Questionnaire: “while watching TV,” “while using a computer at home,” “for other leisure activity time (not including TV or computer use),” “at work,” and “for transport (e.g., in a car, bus, train)” using the response scale of 1 (*no*), 2 (*yes, within 6 months*), 3 (*yes within 1 month*), 4 (*N/A, I don’t watch TV/use a computer at home/I am not currently working*). Responses of N/A (7%) were treated as missing. Items were summed together and a percentage score was calculated ([actual score/greatest possible score not including questions with NA responses]*100). This was done to ensure missing responses did not affect scores.

#### Knowledge

Knowledge of sitting risks was assessed with six items adapted from previous research on the knowledge of health behaviours [30, 31]. Three sitting behaviours were listed: 1) sitting for long periods, 2) sitting for long periods even when regularly active and 3) taking short breaks from sitting by standing or slowly moving around. Participants were asked to rate on a 5 point Likert scale from 1 (*strongly disagree*) to 5 (*strongly agree*) how much they agree that these behaviours increase their risk of (a) chronic disease and (b) depression and overall poor mental health. Exact wording of the 6 items has been published elsewhere [32]. An exploratory factor analysis demonstrated two factors within the knowledge questions with eigenvalues above 1. The first factor was regarding the health risks of sitting for too long, and the second was regarding the health benefits of breaking up sitting time. The first four items (1a, 1b, 2a and 2b) loaded onto the first factor (variance explained = 50%; Cronbach’s alpha = .82) and the last two items (3a and 3b) loaded on to the second factor (variance explained = 20%; Cronbach’s alpha = .76). A knowledge of health risks of sitting score was created by averaging the scores from the four questions loading onto the first factor (range = 1–5) and a knowledge of health benefits of breaking up sitting score was created by averaging the scores from the two questions loading onto the second factor (range = 1–5). It should be noted that currently the evidence on the health impact of sitting time when participants engage in moderate to vigorous intensity physical activity is equivocal with some studies demonstrating that high levels of sitting are associated with poorer health outcomes when accounting for physical activity [1, 33], whilst other studies only show associations between poor health and sitting when participants do not engage in moderate to vigorous physical activity [34, 35]. However, as breaking up sitting with short activity breaks has been found to be beneficial for reducing biomarkers of chronic disease risk [4] we have included the statement ‘Even if I do regular physical activity’ as a measure of knowledge.

#### Likelihood of adherence to messages

After assessing intentions and knowledge, participants were provided with nine messages (seven about reducing sitting time and two about increasing physical activity). Messages were informed by recommendations from Australian heath institutions. This includes recommendations (‘Avoid sitting for more than 2 hours daily in your leisure time’ and ‘Stand and take a break from sitting every 30 minutes’) from the National Heart Foundation of Australia, which is a not-for-profit, non-governmental organisation aimed at improving heart disease prevention and care [36]; the Victorian Health Promotion Foundation which is a Victorian state government organisation aimed at promoting health behaviours in Victoria (‘Stand and take a break from sitting as frequently as you can’) [37]; and the Department of Health, Australian Government (‘Be active at moderate intensity for 30 minutes on most days of the week’ and ‘Move as much as possible on all days of the week’) [38]. Whilst physical activity is a separate behaviour to sitting time, the two physical activity messages were included as a point of reference. For each message, participants were asked to rate how likely they were to adhere to the recommendations using the response scale ranging from 1 (*not at all likely)* to 5 (*very likely*).

### Statistical analyses

For the first objective, to determine self-reported likelihood of adherence, an ordinal regression was conducted with message as the independent variable and self-reported likelihood of adherence as the dependent variable. The reference category was the lowest scoring message for likelihood of adherence: ‘Sit as little as possible on all days of the week’. For the second objective, to test whether self-reported likelihood of adherence to the messages differs as a function of demographic, behavioural (sitting time and physical activity) or psychosocial (knowledge and intentions) characteristics, one ordinal regression was conducted for each message (nine in total). The demographic, psychosocial and behavioural characteristics were entered as independent variables and likelihood of adherence to the message was entered as the dependent variable. It was confirmed that there was no multicollinearity within independent variables (Variance Inflation Factor < 2) prior to conducting the ordinal regression analyses.

## Results

Descriptive statistics of the sample are presented in Table 1. Participants were 55.9 ± 12.9 years old, sat 9.2 ± 3.7 h per day, and engaged in 390.6 ± 391.0 min of physical activity per week, on average. Just over half of participants were women (54%), 36% worked full time, 76% had advanced degree education, and 72% had one or more health conditions.

**Table 1:** Characteristics of Study Sample

Continuous Study Variables	*M*	*SD*
Age (years)	55.86	12.94
Sitting time (hours/day)	9.20	3.72
Physical activity (mins/week)	390.59	391.04
Intentions to limit sitting time (possible range 0–100)	46.63	33.33
Knowledge - risks of sitting (possible range 1–5)	3.55	0.73
Knowledge - benefits of reducing sitting (possible range 1–5)	3.68	0.72
Categorical Study Variables	*n*	%
Gender		
Men	676	46%
Women	784	54%
Education		
Year 12 or less	347	24%
Advanced degree	1113	76%
Employment Status		
Employed full-time	526	36%
Employed part-time	374	26%
Retired/pensioner	432	30%
Home duties, student, & unemployed	128	9%
Health condition presence		
None	1046	72%
One or more	414	28%

*N* = 1460

### Likelihood of adherence to messages

The descriptive statistics for likelihood of adherence to messages are presented in Table 2. Likelihood of adherence differed between the messages (46–83% respectively). The messages ‘Stand and take a break from sitting as frequently as you can,’ and ‘Avoid sitting for more than 10 hours during the entire day’ had the highest levels of likelihood of adherence. The messages ‘Sit as little as possible on all days of the week’ and ‘Avoid sitting for more than 2 hours daily in your leisure time’ had the lowest levels of likelihood of adherence. All messages had a significantly higher self-reported likelihood of adherence when compared to ‘Sit as little as possible on all days of the week’ (*p* < .05).

**Table 2:** A comparison of likelihood of adherence to each message

Message	Likelihood of adherence	
	% Moderately or very likely	OR (95%CI) Reference = message 9
1. Avoid sitting for more than 10 h during the entire day	81.8	7.66 (6.66–8.81)*
2. Stand and take a break from sitting as frequently as you can	82.8	4.07 (3.56–4.65)*
3. Avoid sitting more than 8 h daily in your work	73.2	4.99 (4.36–5.17)*
4. Be active at moderate intensity for 30 min on most days of the week	76.4	3.67 (3.22–4.20)*
5. Move as much as possible on all days of the week	75.9	3.14 (2.75–3.58)*
6. Stand and take a break from sitting every 30 min	75.9	3.19 (2.79–3.64)*
7. Take a short (longer than 1 min) break from sitting every 30 min by standing or doing some activity	75.9	3.06 (2.68–3.50)*
8. Avoid sitting for more than 2 h daily in your leisure time	59.3	1.63 (1.44–1.86)*
9. Sit as little as possible on all days of the week	45.9	1

*p < .05

### Likelihood of adherence as a function of demographic, behavioural and psychosocial characteristics

Additional file 1 presents results of the ordinal regression models testing self-reported likelihood of adherence to the messages as a function of demographic, psychosocial and behavioural characteristics. Results demonstrated that self-reported likelihood of adherence to most messages was highest amongst older participants (except ‘Sit as little as possible on all days of the week’). Women were more likely than men to have a high self-reported likelihood of adherence to most messages (except ‘Be active at moderate intensity for 30 minutes on most days of the week’ and ‘Sit as little as possible on all days of the week’). Respondents who worked part time were more likely than full time workers to have a self-reported likelihood of adherence for ‘Avoid sitting for more than 10 hours during the entire day’ and ‘Avoid sitting more than 8 hours daily in your work.’ Respondents who were retired were more likely than full time workers to have a high self-reported likelihood of adherence to ‘Be active at moderate intensity for 30 min on most days of the week’ but a lower self-reported likelihood of adherence to ‘Avoid sitting more than 8 hours daily in your work’ and ‘Avoid sitting for more than 2 hours daily in your leisure time.’ Respondents who were unemployed were less likely than full time workers to have a self-reported likelihood of adherence to the message ‘Avoid sitting for more than 2 h daily in your leisure time.’ No significant differences were found in self-reported likelihood of adherence as a function of education or disease status.

Self-reported likelihood of adherence to most messages was lower for people with high sitting time (except ‘Stand and take a break from sitting as frequently as you can’). Increased participation in physical activity was associated with increased self-reported adherence to five of the messages, including both messages promoting increased physical activity (‘Be active at moderate intensity for 30 minutes on most days of the week’, ‘Move as much as possible on all days of the week’) and three of the sitting messages. The sitting messages included the message with the highest average self-reported adherence (‘Avoid sitting for more than 10 hours during the entire day’), as well as the two messages with the lowest self-reported adherence (‘Avoid sitting for more than 2 hours daily in your leisure time’ and ‘Sit as little as possible on all days of the week’).

Intentions to sit less was associated with increased likelihood of adherence to most of the messages (except ‘Avoid sitting for more than 10 hours during the entire day’, ‘Avoid sitting more than 8 hours daily in your work’ and ‘Be active at a moderate intensity for 30 minutes on most days of the week’). Knowledge of the benefits of reducing sitting was associated with increased likelihood of adherence to most of the messages (except ‘Be active at moderate intensity for 30 minutes on most days of the week’ and ‘Sit as little as possible on all days of the week’). Knowledge of the health risks of sitting was associated with increased likelihood of adherence to four of the messages, including two messages promoting an increase in positive health behaviours (‘Stand and take a break from sitting every 30 minutes’ and ‘Take a short break from sitting every 30 minutes by standing or doing some activity’), and two negatively framed sitting messages (‘Avoid sitting for more than 2 hours daily in your leisure time’ and ‘Sit as little as possible on all days of the week’).

## Discussion

### Likelihood of adherence to messages

In this study, we investigated Australian adults’ views on a variety of messages aimed to help people reduce sitting time. The high ratings of likelihood of adherence to most of the messages is encouraging in that they demonstrate that recommendations about reducing sitting time can be perceived as achievable by the Australian public.

It has been suggested that public health campaigns are more effective if they promote positively framed messages promoting alternative healthy behaviours rather than negatively framed messages promoting avoidance of unhealthy behaviours [12, 39], and this is partially supported by the present study in that the two messages with the lowest self-reported likelihood of adherence promoted avoidance of sitting. One of the two messages with the highest self-reported likelihood of adherence was, however, also framed as the avoidance of sitting (‘Avoid sitting for more than 10 hours during the entire day’). This could also be due to the message being perceived as highly achievable, as 10 h of sitting allows people to sit for the majority of their waking hours and is greater than the average daily sitting time in Australia [3]. The positive messages promoting physical activity scored fourth and fifth out of nine on self-reported likelihood of adherence. These messages scored higher than the message about taking a break from sitting to be active, but may not be effective at reducing sedentary behaviour. Past research has demonstrated that focusing on increasing physical activity alone has minimal incidental effect on sedentary behaviour [40].

It is not surprising that the messages with low ratings of likelihood of adherence were context specific (i.e. work and leisure time). The low ratings are likely to be due to these messages only being relevant to specific population groups (e.g. people who work full time) and only in specific contexts (e.g. during work hours). Public health campaigns targeting the general population through mass media should therefore avoid context specific messages. The low ratings of likelihood of adherence to the context specific messages could also be due to the difficultly in reducing sitting time in these contexts (e.g., at work). Messages targeting these areas may need to focus on more achievable strategies such as interrupting sitting by having brief standing or walking breaks, rather than reducing overall sitting time which may not be possible due to work requirements.

The significantly lower self-rated likelihood of adherence to the message ‘Sit as little as possible on all days of the week’ compared to all other messages could be due to it being too broad. Research demonstrates that people are more likely to achieve goals that are detailed and specific [41]. It should, however, be noted that the message with the highest ratings of likelihood of adherence (‘Stand and take a break from sitting as frequently as you can’) was not as specific as other messages promoting breaks with lower ratings of likelihood of adherence (e.g. ‘Stand and take a break from sitting every 30 minutes’). This may be because of the perception that standing every 30 min does not seem achievable. Research using objective measures of sitting has demonstrated that sitting events greater than 30 min in duration are common in most people [42]. Perceived ability to adhere to a message is closely related to self-efficacy, or the belief that one can perform a particular behaviour [43, 44]. Self-efficacy is a predictor of behaviour change which may explain the lower ratings of likelihood of adherence for the more challenging messages [43, 44]. Therefore specific messages promoting an achievable frequency of sitting breaks such as every hour may be a good starting point for health promotion campaigns.

It is positive that the Australian Government Department of Health and Aging promotes taking breaks from sitting as often as possible as this has the highest rating of likelihood of adherence, however the department also promotes sitting as little as possible which has the lowest rating of likelihood of adherence [5]. Our results suggest that a more specific but achievable message (e.g. sitting less than 10 h in the entire day) may be more effective at engaging the public than the message to sit as little as possible. However, the challenge is to present an achievable message that is in line with the evidence that sitting should be kept to less than 8 h a day [1–3].

### Likelihood of adherence as a function of demographic, behavioural and psychosocial characteristics

Likelihood of adherence to most messages was positively associated with age, which may be due to older adults being more interested in their health as they are at a greater risk of chronic disease and functional limitations arising from high sitting and inactivity [45]. Further, older adults, particularly those who are retired are more likely to have greater flexibility with their time and therefore have less barriers to changing their sitting behaviour [46]. Compared to women, men were less likely to have a high self-reported likelihood of adherence to most messages. This is in line with past research findings showing that men are less likely to be interested in improving their health behaviours than women [47]. This association was however not seen for the message, ‘Be active at moderate intensity’ which may be due to the higher levels of physical activity in males [48].

People who are unemployed or retired were less likely to have a high self-reported likelihood of adherence to the message ‘Avoid sitting for more than 2 hours daily in your leisure time.’ This is probably because they have more leisure time hours in a day making it more difficult to adhere to this message [49, 50]. Conversely retired participants were more likely to have a higher self-reported likelihood of adherence to the message about being active at a moderate intensity. This finding may be a result of the fact that retired adults have more free time to engage in physical activity [46, 50]. Messages promoting physical activity could therefore be used to engage retired adults, however, it should still be made clear that prolonged sitting time leads to negative health outcomes even when physically active [1, 51].

Self-reported likelihood of adherence to the message ‘Stand and take a break’ was the only message not negatively associated with sitting time and had a higher overall rating of likelihood of adherence. Therefore, to engage people who are sitting a lot, health promotion campaigns could use this message promoting standing breaks. In light of recent evidence demonstrating greater health benefits of walking breaks compared to standing breaks, campaigns should also consider the use of messages promoting walking breaks [52]. As expected, reported likelihood of adherence to the messages promoting physical activity were positively associated with physical activity. The messages about reducing sitting time or taking sitting breaks may therefore be more engaging to those with lower levels of physical activity. It is however important to also address their low levels of physical activity to reduce their risk of chronic disease [53].

As expected, knowledge of sitting risks was positively associated with the self-reported likelihood of adherence to most messages and knowledge of the benefits of reducing sitting time was positively associated with several of the messages. This is in line with past research which highlights knowledge as an important in first step getting a person to engage with behaviour change messages [54] and demonstrates the importance of education. Those with lower intentions to reduce their sitting time were less likely to feel as though they could follow most messages. These results support previous research showing that mass media public health campaigns serve as a guide for people whom are already motivated, rather than for those who are not yet motivated [55].

### Strengths and limitations

This study serves to guide the framing and content of public health recommendations about reducing sitting time. Although the sample had a high level of sitting time, they were relatively active and more research is necessary to test whether these results generalize across different populations. The study was a web-based cross-sectional survey, recording participants’ perceptions of how likely they would follow the messages. Research has shown that people’s insights of their future behaviour can be poor [56]. Therefore causation or how messages impact behaviour change cannot be determined. Future experimental research testing the efficacy of the messages in changing people’s sitting time will be an important next step toward developing persuasive public health messages. Self-report surveys can lead to bias and the low percentage (42%) of the panel who completed the survey may have influenced the representativeness of the sample. Lastly, the study was conducted in Australia with Australian participants and messages based on Australian sitting time messages. This limits generalisability of the results to other countries. However, the findings can still be used as the basis of further work investigating how sitting messages are received in different population groups and in different countries.

## Conclusions

The results of this study provide valuable information for the development of public health messages about reducing sitting time. Sitting messages may be more effective at changing behaviour across a large population if they are achievable, specific and framed as a promotion of healthy alternatives to sitting, such as standing or being active. Messages promoting an increase in positive health behaviours (e.g. sitting breaks) may be more likely to engage people with high sitting behaviour and messages promoting physical activity may be more likely to engage males and retired adults.

## Additional file

Additional file 1: How likelihood of adherence differs as a function of demographic, psychosocial and behavioural characteristics. (DOCX 18 kb)

## Data Availability

The datasets used and/or analysed during the current study are available from the corresponding author on reasonable request.
