# Supplementary material for: Should I sit or stand: likelihood of adherence to messages about reducing sitting time
Source: BMC Public Health. 2019 Jul 3;19:871. doi: 10.1186/s12889-019-7189-z (PMC6610814; doi:10.1186/s12889-019-7189-z)
Supplement: Supplementary file 1 — How likelihood of adherence differs as a function of demographic, psychosocial and behavioural characteristics. (DOCX 18 kb) [file 12889_2019_7189_MOESM1_ESM.docx]

*How likelihood of adherence differs as a function of demographic, psychosocial and behavioural characteristics.*

|  | **Likelihood of adherence to messages** | | | | | | | | |
| --- | --- | --- | --- | --- | --- | --- | --- | --- | --- |
|  | 1. Avoid sitting for more than 10 hours during the entire day  **OR (95% CI)** | 2. Stand and take a break from sitting as frequently as you can  **OR (95% CI)** | 3. Avoid sitting more than 8 hours daily in your work  **OR (95% CI)** | 4. Be active at moderate intensity for 30 minutes on most days of the week  **OR (95% CI)** | 5. Move as much as possible on all days of the week  **OR (95% CI)** | 6. Stand and take a break from sitting every 30 minutes  **OR (95% CI)** | 7. Take a short break from sitting every 30 minutes by standing or doing some activity  **OR (95% CI)** | 8. Avoid sitting for more than 2 hours daily in your leisure time  **OR (95% CI)** | 9. Sit as little as possible on all days of the week  **OR (95% CI)** |
| **Age (years)** | **1.02 (1.01-1.04)*** | **1.02 (1.01-1.03)*** | **1.01 (1.00-1.02)*** | **1.03 (1.01-1.04)*** | **1.03 (1.02-1.04)*** | **1.02 (1.01-1.03)*** | **1.02 (1.01-1.03)*** | **1.02 (1.01-1.03)*** | 1.00 (0.99-1.01) |
| **Sitting time (hours/day)** | **0.86 (0.83-0.88)*** | 0.98 (0.95-1.00) | **0.88 (0.86-0.91)*** | **0.95 (0.92-0.98)*** | **0.93 (0.90-0.95)*** | **0.95 (0.93-0.98)*** | **0.97 (0.94-1.00)*** | **0.90 (0.88-0.93)*** | **0.93 (0.90-0.95)*** |
| **Physical activity (mins/week)** | **1.00 (1.00-1.00)*** | 1.00 (1.00-1.00) | 1.00 (1.00-1.00) | **1.00 (1.00-1.00)*** | **1.00 (1.00-1.00)*** | 1.00 (1.00-1.00) | 1.00 (1.00-1.00) | **1.00 (1.00-1.00)*** | **1.00 (1.00-1.00)*** |
| **Intentions** | 1.01 (1.00-1.01) | **1.01 (1.00-1.01)*** | 1.00 (0.99-1.01) | 1.01 (1.00-1.01) | **1.01 (1.00-1.01)*** | **1.01 (1.00-1.01)*** | **1.01 (1.00-1.02)*** | **1.01 (1.00-1.01)*** | **1.02 (1.01-10.2)*** |
| **Knowledge- benefits of reducing sitting** | **1.16 (1.07-1.26)*** | **1.24 (1.15-1.34)*** | **1.14 (1.05-1.23)*** | 1.08 (1.00-1.16) | **1.12 (1.04-1.20)*** | **1.16 (1.08-1.26)*** | **1.18 (1.10-1.27)*** | **1.11 (1.03-1.20)*** | 1.05 (0.97-1.13) |
| **Knowledge- sitting risks** | 1.00 (0.96-1.05) | 1.01 (0.97-1.05) | 1.02 (0.98-1.06) | 1.02 (0.98-1.06) | 1.02 (0.98-1.06) | **1.06 (1.02-1.10)*** | **1.06 (1.02-1.10)*** | **1.06 (1.02-1.10)*** | **1.09 (1.05-1.13)*** |
| **Gender** |  |  |  |  |  |  |  |  |  |
| Men | **0.73 (0.58-0.92)*** | **0.69 (0.56-0.85)*** | **0.79 (0.64-0.98)*** | 1.05 (0.85-1.31) | **0.68 (0.56-0.84)*** | **0.72 (0.58-0.89)*** | **0.73 (0.59-0.90)*** | **0.74 (0.61-0.91)*** | 0.94 (0.77-1.15) |
| Women | 1 | 1 | 1 | 1 | 1 | 1 | 1 | 1 | 1 |
| **Education** | | | | | | | | | |
| Year 12 or less | 0.90 (0.70-1.17) | 1.06 (0.84-1.34) | 0.87 (0.68-1.11) | 1.12 (0.88-1.42) | 1.21 (0.96-1.53) | 1.01 (0.80-1.27) | 0.93 (0.74-1.17) | 1.18 (0.94-1.47) | 1.19 (0.95-1.49) |
| Advanced degree | 1 | 1 | 1 | 1 | 1 | 1 | 1 | 1 | 1 |
| **Employment** | | | | | | | | | |
| Unemployed | 1.46 (0.96-2.21) | 0.92 (0.64-1.35) | 1.08 (0.73-1.60) | 1.28 (0.88-1.87) | 0.93 (0.64-1.34) | 1.13 (0.78-1.64) | 1.09 (0.75-1.57) | **0.60 (0.42-0.85)*** | 1.12 (0.78-1.61) |
| Retired/pensioner | 1.25 (0.89-1.76) | 0.80 (0.59-1.09) | **0.60 (0.43-0.82)*** | **1.41**  **(1.03-1.93)*** | 0.86 (0.64-1.17) | 0.91 (0.68-1.24) | 0.89 (0.66-1.20) | **0.56 (0.42-0.76)*** | 1.05 (0.78-1.40) |
| Employed part-time | **1.20 (1.12-2.20)*** | 0.92 (0.70-1.20) | **1.63 (1.22-2.18)*** | 1.19  (0.91-1.56) | 0.92 (0.71-1.20) | 0.92 (0.71-1.20) | 0.92 (0.71-1.19) | 0.95 (0.73-1.23) | 1.00 (0.77-1.29) |
| Employed full-time | 1 | 1 | 1 | 1 | 1 | 1 | 1 | 1 | 1 |
| **Disease status** | | | | | | | | | |
| One or more | 1.03 (0.79-1.35) | 0.95 (0.75-1.21) | 0.85 (0.67-1.08) | 0.99  (0.78-1.26) | 0.89 (0.71-1.12) | 0.97 (0.77-1.22) | 0.90 (0.71-1.13) | 0.87 (0.69-1.09) | 0.83 (0.67-1.04) |
| None | 1 | 1 | 1 | 1 | 1 | 1 | 1 | 1 | 1 |

**p*<.05
